# Effects of Cooling Media on Microstructure and Mechanical Properties in Friction Stir Welded SA516 Gr.70 Cryogenic Steel Joints

**DOI:** 10.3390/ma17184661

**Published:** 2024-09-23

**Authors:** Xiuying Wang, Yu Wang, Jiujun Xu, Juncai Sun, Yuqian Wang, Guangming Xie

**Affiliations:** 1 Institute of Materials and Technology, Dalian Maritime University, Dalian 116026, China; janewxy@cpm.com.cn (X.W.); jjxu@dlmu.edu.cn (J.X.); 2 Challenge Petrochemical Machinery Corporation of Maoming (CPM), Maoming 525024, China; wangyu@cpm.com.cn; 3 State Key Laboratory of Rolling and Automation, Northeastern University, Shenyang 110819, China; yqwangneu@126.com

**Keywords:** friction stir welding, cryogenic steel, cooling media, microstructure evolution, properties

## Abstract

SA516 Gr.70 steels were welded by friction stir welding (FSW) under various media of air, water, and water + CO_2_ cooling, and the effect of the cooling media on the microstructure and mechanical properties of joints was systematically analyzed. The nugget zone (NZ) under the air-cooling condition contained coarse bainite + martensite. Martensite was obtained by decreasing the cooling media temperature. Furthermore, tensile fracturing of the joints occurred in the basal metal (BM), and the ultimate tensile strength of the joints under various cooling media was similar to that of the BM. However, with decreasing cooling media temperature, the total elongation of the joints noticeably increased. Good strength (545 MPa) and elongation (16.8%) were obtained in the joints under the water + CO_2_ cooling condition since the fine martensite microstructure enhanced the plastic deformation capacity of the joints. In addition, in the NZ under water + CO_2_ cooling condition, good toughness of 110 J/cm^2^ was obtained due to a high fraction of high-angle boundaries and fine martensite.

## 1. Introduction

Typically, the normalized microstructure of SA516 Gr.70 cryogenic steel contains pearlite and ferrite, possessing excellent properties which have great application potential in the field of cryogenic vessels [1]. Currently, SA516 Gr.70 cryogenic steel is mainly welded by fusion welding in engineering structures [2,3]. Defects such as porosity, hot cracking, and oxidation tend to appear in the weld. In addition, the coarse grain of joints seriously degrades the mechanical properties [3,4]. Therefore, how to further improve the welding quality of SA516 Gr.70 cryogenic steel to contribute to the reliability of pressure vessels in the harsh environment of safe operations has become a key and urgent issue in engineering.

Friction stir welding (FSW), as a new solid-phase joining technology, is considered to be the most revolutionary welding technology of the 21st century [5]. During FSW, a remarkable dynamic recrystallization occurs in the nugget zone (NZ) under the coupling action of heat and strong plastic deformation, and fine recrystallized grains are obtained. In this case, a reliable connection between workpieces is realized. Furthermore, the microstructural coarsening of the HAZ is effectively inhibited depending on the low heat input of FSW, thereby improving the toughness of the HAZ. Furthermore, compared with arc welding, FSW can avoid cracks, porosity, and segregation, obtaining high-quality joints.

At present, FSW is mainly used to join aluminum alloy, which is widely used in the aerospace, automobile, and rail transportation fields [6,7]. However, there have been few studies on FSW with cryogenic steel, especially on the microstructure and properties of the joint. Previously, we used FSW to join cryogenic steel plates under various rotation rates and found that with increasing rotational rate, the microstructure of the NZ changed from bainite + martensite to martensite, and the grain was obviously coarsened, resulting in a decrease in toughness in the joints [8]. To improve the toughness of joints, post-welding tempering was applied to FSW SA516 Gr.70 steel joints. In the annealed FSW joints with tempered martensite, dislocation density decreased, and carbide precipitating along grain boundaries was found, which significantly improved the toughness and elongation of the joints [9].

Recently, it has been shown that rapid external cooling during FSW reduces peak temperatures and the duration of high temperatures, thus inhibiting grain coarsening [10,11]. Xue et al. [10] performed FSW on X80 steel under water-cooling conditions and suggested that a fine martensite lath and ferrite were obtained. Similarly, Imam et al. [11] carried out a study of FSW of media carbon steel under liquid CO_2_ cooling conditions and pointed out that the grain of the NZ was obviously refined, and the strength and elongation of the joints were significantly improved. Therefore, FSW with an external cooling medium is expected to further optimize the properties in the joint. However, there are few reports about the effect of cooling media on the microstructural evolution and mechanical properties of FSW SA516 Gr.70 cryogenic steel joints.

In this study, SA516 Gr.70 cryogenic steels were welded via FSW under various cooling media of air, water, and water + CO_2_. The microstructure of each subzone in the joints under different cooling media was analyzed in detail, and the hardness distribution, tensile properties, and impact properties of the joints were also tested. The effects of the cooling media on the microstructural evolution and mechanical properties in the joints were investigated. Finally, the relationship between the microstructure and mechanical properties of the joints was established.

## 2. Experimental Procedure

According to the ASME standard, SA516 Gr.70 steel is a form of cryogenic-temperature steel, with excellent toughness and elongation. Therefore, SA516 Gr.70 steel (~2.5 mm) with pearlite and ferrite was considered as a base metal (BM), whose chemical composition is Fe-0.16C-0.32Si-1.47Mn (wt.%). The cryogenic steel was connected via FSW at a rotation rate of 400 rpm and a welding speed of 100 mm/min under different cooling media of air, water, and mixed water and solid CO_2_ (water + CO_2_). During welding, the whole plate was welded in the cooling media. Furthermore, during FSW, the tool had a tilt angle of 3° from the normal direction of SA516 Gr.70 steel plates, and the plunge depth of the tool shoulder was ~0.2 mm. A welding tool with a concave shoulder of 12 mm in length and a stirring pin of 2 mm in length was adopted. Therefore, SA516 Gr.70 steel plates were partially penetrated. In addition, the material of the stirring pin was W-25Re (wt.%) alloy.

The microstructure of the joints was analyzed by a Leica optical microscope (OM, Leica, Germany), scanning electron microscope (SEM, Zeiss Gemini 300, Jena, Germany) (SEM) with electron backscatter diffraction (EBSD, Oxford Instruments Symmetry, Abingdon, UK), and transmission electron microscope (TEM, FEI-Tecnai G2-F20, Thermo Fisher Scientific, Waltham, MA, USA). SEM and OM samples were prepared by polishing and etching with ~8% nital. The samples for EBSD were electro-polished with a 10% perchloric acid + 90% ethanol solution at 22 V for 25 s. Based on the EBSD data, AztecCrystal^®^ 5.0 software was used to analyze the grain size of each subzone in the joints under various cooling media. TEM samples were prepared via twin-jet polishing at −25 °C.

**Figure 1:**
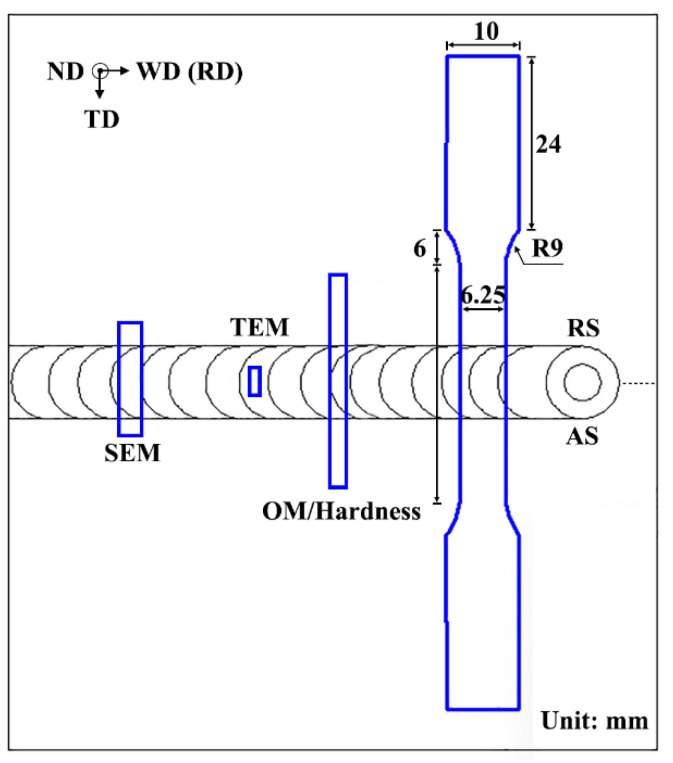
Schematics of sampling location of the microstructure and properties.

The Vickers hardness in the HAZ and NZ was analyzed by a Future-Tech FM-700 micro-hardness machine (Kawasaki, Japan). The tensile properties of the transverse joints were tested by a tensile machine (GOTECH, AI-7000-LAU10, Taiwan, China) at a constant tensile rate of 2 mm/min. The size of the microstructure and tensile samples are shown in Figure 1. Furthermore, the Charpy V-notch impact energy of the HAZ and NZ was evaluated by an Instron machine (Norwood, MA, USA) at −46 °C. The 2 mm thick samples were applied to analyze the toughness of the joint.

## 3. Results and Discussion

### 3.1. Microstructure Evolution of the Joint under Various Cooling Media

**Figure 2:**
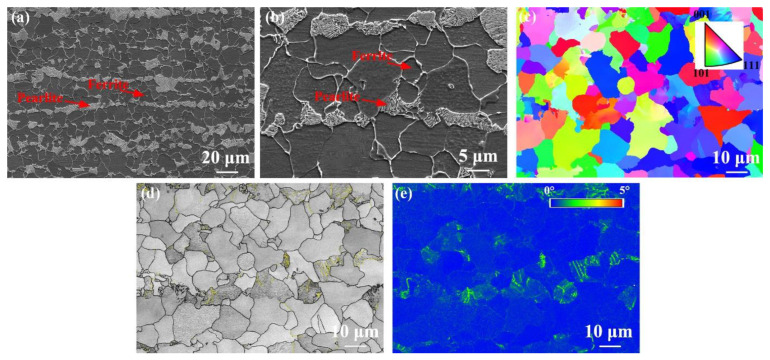
Microstructure in the BM. (**a**,**b**) SEM, (**c**) inverse pole figure (IPF), (**d**) grain boundary map, (**e**) KAM map.

Figure 2 presents the microstructure images of the BM. Pearlite and ferrite were observed in the BM (Figure 2a,b). Figure 2c–e show the EBSD results of the BM. The BM presented a random orientation distribution, as shown in Figure 2c. In Figure 2d, black lines and yellow lines represent high-angle boundary (HAB) and low-angle boundary (LAB), respectively. The misorientation angles of HAB and LAB are 2~15 and ≥15, respectively. In the BM, the fraction of LAB and HAB was 15% and 85%, respectively. Obviously, in the BM, a low fraction of LAB was found, which should be attributed to the occurrence of recovery during normalizing. Usually, the low kernel average misorientation (KAM) values represent a low dislocation density [12]. The average KAM value in the BM was 0.31°. Clearly, a low KAM value was found in the BM, corresponding to a low dislocation density (Figure 2e).

**Figure 3:**
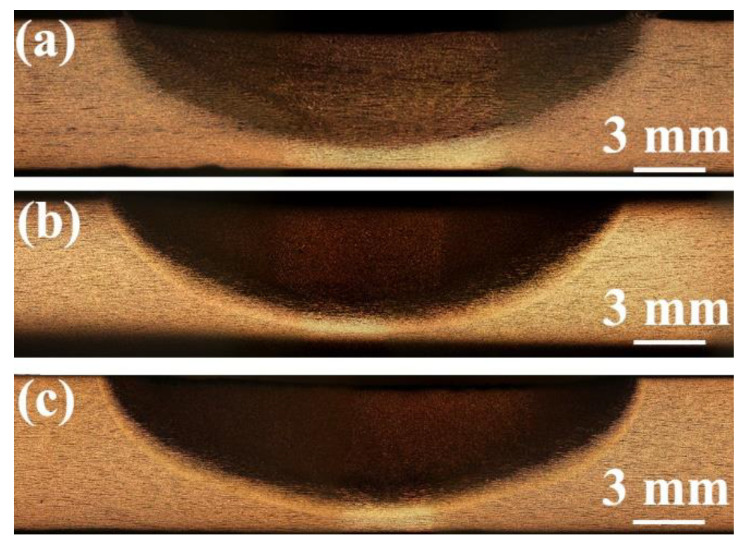
Macrographs of SA516 Gr.70 steel joints under various media. (**a**) Air cooling, (**b**) water cooling, (**c**) water + CO_2_ cooling.

Figure 3 presents the macrographs in FSW cryogenic steel joints under various media conditions. Obviously, the joint presented a basin joint and had no obvious defects. The NZ, heat-affected zone (HAZ), and BM can be clearly distinguished in the joint. The range of the NZ under various media conditions had no obvious change. However, the width of the HAZ under water cooling and water + CO_2_ cooling decreases compared to that under the air condition owing to the low peak temperature and short residence time at high temperatures caused by the rapid post-welding cooling rate.

**Figure 4:**
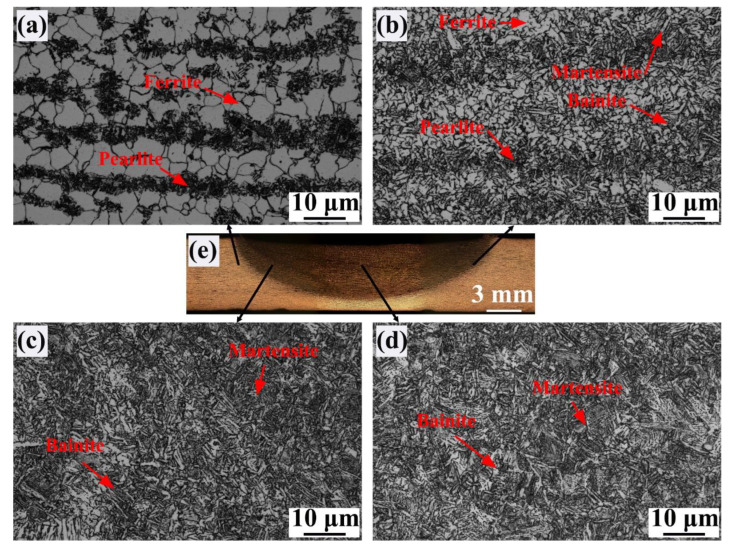
OM images in the SCHAZ (**a**), ICHAZ (**b**), FGHAZ (**c**), NZ (**d**), macroscopic joint (**e**) under air cooling.

Figure 4 shows OM images of FSW joints under air-cooling conditions. The NZ contained coarse bainite + lath martensite (Figure 4d). During FSW, the high peak temperature and strong plastic deformation in the NZ are good for the presence of austenitic recrystallization. However, the long residence time at high temperatures and slow cooling rate of the NZ lead to significant coarsening of austenite. Finally, coarse bainite + martensite was obtained. Generally, the HAZ can be subdivided into fine-grain HAZ (FGHAZ), inter-critical HAZ (ICHAZ), and sub-critical HAZ (SCHAZ), whose peak temperatures are located at > A_c3_, A_c1_–A_c3,_ and < A_c1_, respectively [13]. The microstructure in the SCHAZ contained ferrite and pearlite, similar to that in the BM (Figure 4a). However, in the ICHAZ, besides ferrite and pearlite, bainite and martensite were also observed (Figure 4b). The FGHAZ consisted of bainite and martensite (Figure 4c). Therefore, with regard to the SCHAZ, the microstructure of the BM experiences tempering. However, FSW had low heat input, so there were no significant changes in the microstructure in the SCHAZ relative to that of the BM. The peak temperature in the ICHAZ is at A_c1_–A_c3_, so part of pearlite in the BM undergoes austenitizing. Subsequently, austenite changes into bainite + martensite during cooling with a high cooling rate. In addition, the remanent ferrite and pearlite are reserved. In this case, ferrite + pearlite + bainite + martensite was gained. The FGHAZ was completely austenitized during FSW since the peak temperature of the FGHAZ is higher than A_c3_. Therefore, bainite + martensite was obtained with a rapid cooling rate.

**Figure 5:**
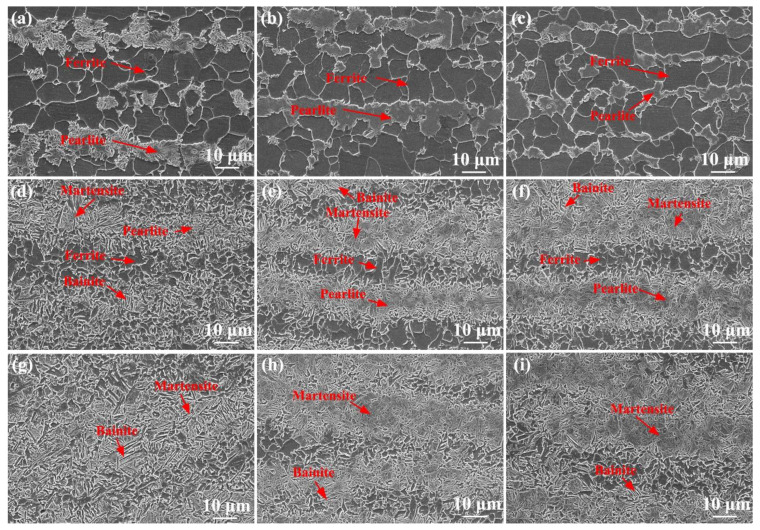
SEM images in the SCHAZ (**a**–**c**), ICHAZ (**d**–**f**), FGHAZ (**g**–**i**); (**a**,**d**,**g**) air cooling, (**b**,**e**,**h**) water cooling, (**c**,**f**,**i**) water + CO_2_ cooling.

Figure 5 shows SEM images of the HAZ under various cooling conditions. Pearlite and ferrite were obtained in the SCHAZ under various cooling media (Figure 5a–c). Under the air-cooling condition, C in pearlite of the SCHAZ diffuses, while with decreasing cooling media temperature, the diffusion of C in pearlite is inhibited, and the grain size is refined because of the decrease in high-temperature residence time, reducing the degree of the tempering. Therefore, the microstructure in the SCHAZ under water + CO_2_-cooling conditions is similar to that of the BM. In the ICHAZ under various cooling media, ferrite + pearlite + bainite + martensite was observed (Figure 5d–f). As the cooling media temperature decreases, martensite content in the ICHAZ increases, which was attributed to the high post-welding cooling rate, promoting martensitic transformation. In addition, the grains in the ICHAZ are refined by decreasing the cooling media temperature. The FGHAZ under various cooling media mainly contained martensite + bainite microstructure, as shown in Figure 5e–i. As the cooling media temperature decreased, martensite content in the FGHAZ noticeably increased, and the grain size decreased.

**Figure 6:**
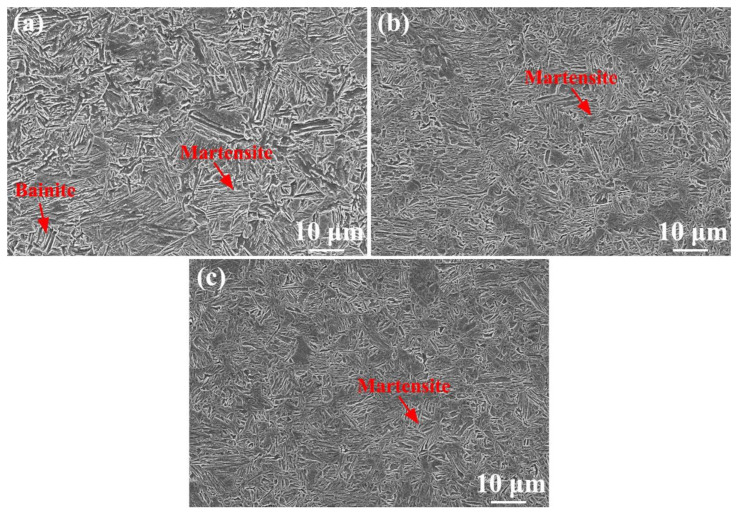
Images of the NZ under air cooling (**a**), water cooling (**b**), water + CO_2_ cooling (**c**).

Figure 6 shows the SEM microstructure in the NZ under different cooling media. It was found that with decreasing cooling media temperature, the grain size decreased due to lower peak temperature, effectively inhibiting grain growth (Figure 6a–c). In the NZ under air-cooling conditions, bainite + martensite was observed (Figure 6a). Martensite content in the NZ increased obviously with decreasing cooling media temperature. Under water + CO_2_ cooling, the NZ mainly contained martensite. In this case, a large amount of low-temperature phase transformation products were obtained in the NZ by decreasing the cooling media temperature due to the increase in undercooling.

**Figure 7:**
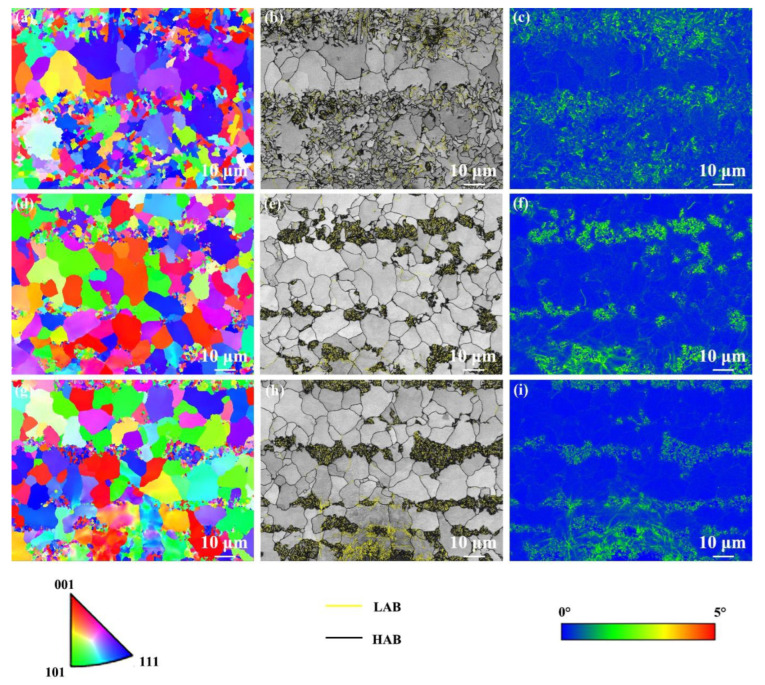
EBSD results of the SCHAZ under various cooling media. (**a**,**d**,**g**) IPF maps, (**b**,**e**,**h**) grain boundaries distribution maps, (**c**,**f**,**i**) KAM maps; (**a**–**c**) air cooling, (**d**–**f**) water cooling, (**g**–**i**) water + CO_2_ cooling.

Figure 7 shows the EBSD results of the SCHAZ under different cooling media. It was detected that the SCHAZ presented a random orientation distribution (Figure 7a,d,g). The grain size in the SCHAZ at air cooling, water cooling, and water + CO_2_ cooling was 4.0, 3.1, and 2.7 μm, respectively. Obviously, the grain in the SCHAZ was refined with the decrease in the cooling media temperature. In contrast, the grain in the SCHAZ in fusion welded cryogenic steel joints grew obviously [8]. The fraction of HAB in the SCHAZ under air cooling, water cooling, and water + CO_2_ cooling was 76%, 74%, and 75%, respectively. Clearly, the fraction of HAB in the SCHAZ under various cooling media has no obvious change, which should be due to the low heat input. In the SCHAZ under different cooling media, the distribution of KAM values was not uniform because of pearlite with high KAM values and ferrite with low KAM values (Figure 7c,f,i).

**Figure 8:**
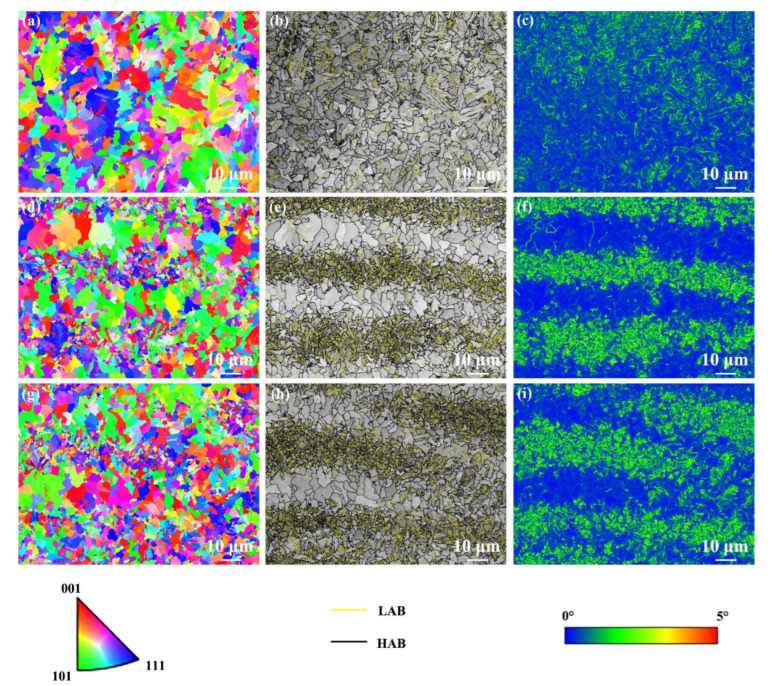
EBSD results of the ICHAZ under various cooling media. (**a**,**d**,**g**) IPF maps, (**b**,**e**,**h**) grain boundary distribution maps, (**c**,**f**,**i**) KAM maps; (**a**–**c**) air cooling, (**d**–**f**) water cooling, (**g**–**i**) water + CO_2_ cooling.

Figure 8 shows the EBSD results of the ICHAZ under various cooling media. The ICHAZ under various cooling media exhibited random orientation distribution (Figure 8a,d,g). The grain size in the ICHAZ at air cooling, water cooling, and water + CO_2_ cooling was 3.2, 2.1 and 1.9 μm, respectively. Obviously, the grain in the ICHAZ was refined under the external rapid cooling media. The microstructure distribution in the ICHAZ under air cooling conditions was relatively uniform, whereas the microstructure of the ICHAZ under water-cooling and water + CO_2_-cooling conditions was still alternately distributed. This result is attributed to the peak temperature of the ICHAZ under air cooling during FSW being higher, and more pearlite and ferrite transform into austenite. Furthermore, the fraction of LAB in the ICHAZ under air-cooling, water-cooling, and water + CO_2_-cooling conditions was 30%, 33%, and 35%, respectively. Obviously, the fraction of LAB in the ICHAZ increases with the decrease in the cooling media temperature. As the cooling media temperature decreases, martensite content increases, increasing the fraction of LAB. In addition, the KAM distribution of ICHAZ under air-cooling conditions was relatively uniform, while the KAM distribution of ICHAZ under water cooling and water + CO_2_ cooling was very uneven (Figure 8c,f,i). The average KAM values of the ICHAZ under air cooling, water cooling, and water + CO_2_ cooling were 0.6°, 0.7°, and 0.8°, respectively. It can be found that the average KAM value in the ICHAZ increases monotonously with decreasing cooling media temperature, corresponding to the appearance of a high fraction of martensite. This result is consistent with the change in LAB. The ICHAZ exhibited a high KAM value relative to that of the SCHAZ, irrespective of the cooling media, due to the existence of martensite + bainite.

**Figure 9:**
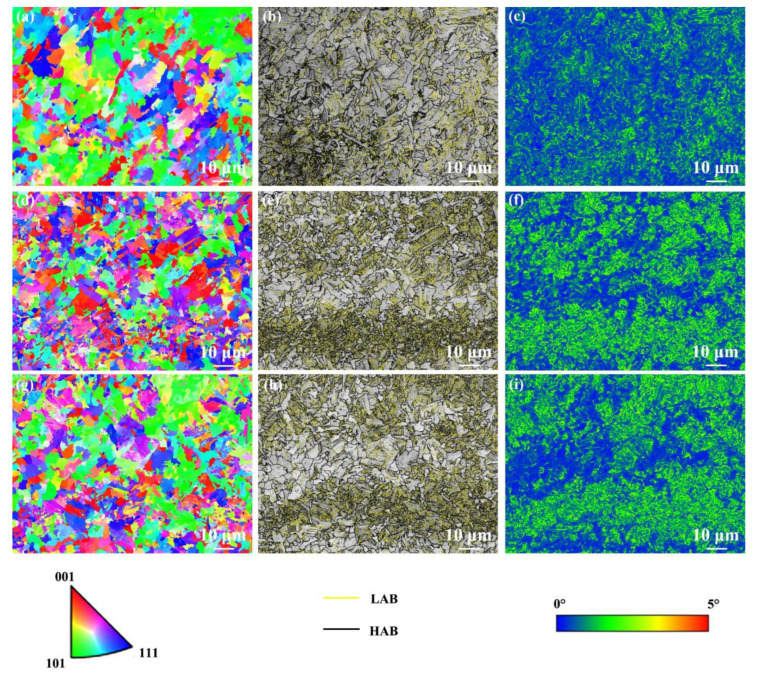
EBSD results of the FGHAZ under various cooling media. (**a**,**d**,**g**) IPF maps, (**b**,**e**,**h**) grain boundary distribution maps, (**c**,**f**,**i**) KAM maps; (**a**–**c**) air cooling, (**d**–**f**) water cooling, (**g**–**i**) water + CO2 cooling.

Figure 9 shows the EBSD results of the FGHAZ under various cooling media. The FGHAZ under various cooling media exhibited random orientation distribution (Figure 9a,d,g). The grain size in the FGHAZ in air-cooling, water-cooling, and water + CO_2_-cooling conditions was 3.4, 2.3, and 2.0 μm, respectively. It can be found that as the cooling media temperature decreases, the grain of FGHAZ is refined because of the lower peak temperature. Moreover, the fraction of HAB of FGHAZ under air cooling, water cooling, and water + CO_2_ cooling was 55%, 56%, and 58%, respectively. Obviously, as the cooling media temperature decreases, the fraction of HAB in the FGHAZ increases slightly, which should be due to the grain refinement under the external rapid cooling media. The average KAM values in the FGHAZ under air cooling, water cooling, and water + CO_2_ cooling were 0.69°, 0.96°, and 0.97°, respectively. Obviously, the FGHAZ under water-cooling and water + CO_2_-cooling conditions exhibited a higher KAM value than that under air cooling since martensite content increased under external rapidly cooling media (Figure 9c,f,i). In addition, the KAM value of FGHAZ under various cooling media increased significantly compared with that of the SCHAZ and ICHAZ.

**Figure 10:**
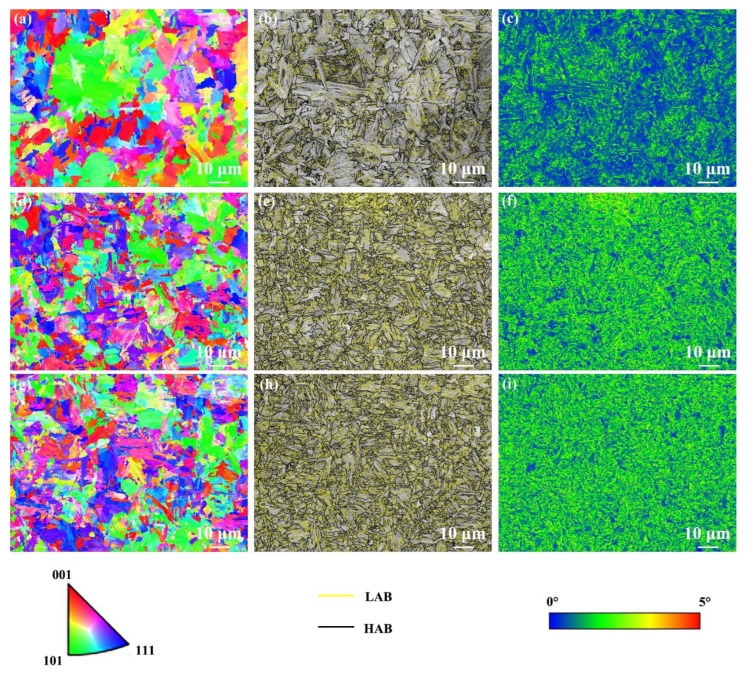
EBSD results of the NZ under various cooling media. (**a**,**d**,**g**) IPF maps, (**b**,**e**,**h**) grain boundary distribution maps, (**c**,**f**,**i**) KAM maps; (**a**–**c**) air cooling, (**d**–**f**) water cooling, (**g**–**i**) water + CO_2_ cooling.

Figure 10 shows EBSD results of the NZ under various cooling media. The NZ under various cooling media presented a random orientation distribution (Figure 10a,d,g). The grain size in the NZ under air cooling, water cooling, and water + CO_2_ cooling was 3.5, 2.5, and 2.2 μm, respectively. It can be found that the grains in the NZ are refined with decreasing cooling media temperature, especially under water + CO_2_-cooling conditions owing to the lower peak temperature. Moreover, the fraction of HAB in the NZ under air cooling, water cooling, and water + CO_2_ cooling is 49%, 48%, and 49%, respectively. Generally, grain refinement tends to increase the fraction of HAB. Interestingly, the fraction of HAB in the NZ under various cooling media is similar. As the cooling media temperature decreases, the displacive phase transformation during post-weld cooling produces a large amount of dislocation, increasing the fraction of LAB. However, the total fraction of LAB to HAB is constant (100%). Finally, the NZ exhibits a similar fraction of HAB under the integration of dislocation substructure and grain size. The KAM values of the FGHAZ under air cooling, water cooling, and water + CO_2_ cooling were 0.86°, 1.34°, and 1.35°, respectively. Obviously, the NZ under water-cooling and water + CO_2_-cooling conditions exhibit a higher KAM value than that under air cooling since martensite content increases under external rapid cooling media (Figure 10c,f,i). In addition, compared with the case of the HAZ, the KAM value of the NZ under various cooling media increased significantly.

**Figure 11:**
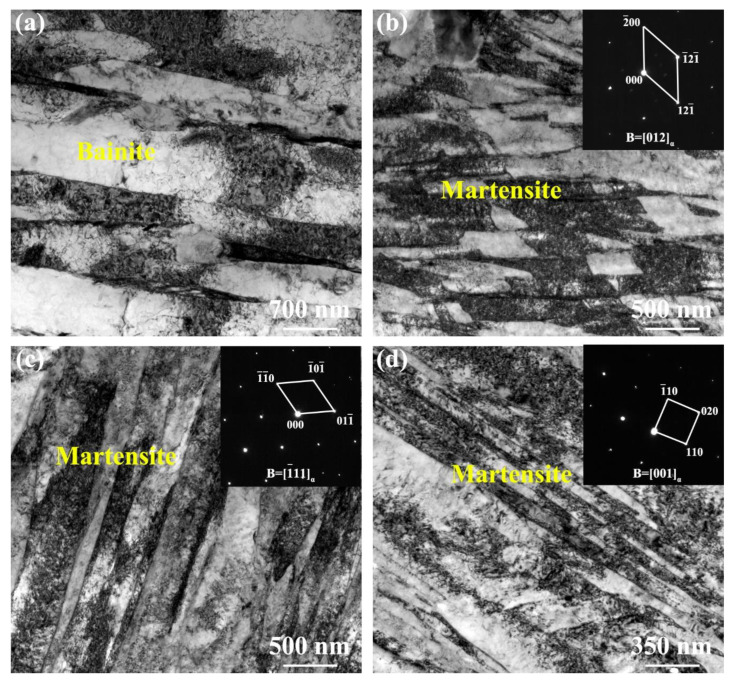
TEM microstructure in the NZ under air cooling (**a**,**b**), water cooling (**c**), water + CO_2_ cooling (**d**).

To further analyze the microstructure characteristics of the NZ under various cooling media, TEM measurement was carried out, as shown in Figure 11. Under air-cooling conditions, the NZ contained bainite + martensite containing a large amount of dislocations, as shown in Figure 11a,b. The microstructure of the NZ under water + CO_2_-cooling condition mainly consisted of martensite (Figure 11d). This is due to the low cooling media temperature leading to higher undercooling, promoting the occurrence of martensitic transformation [10,11]. The martensite lath in the NZ under water + CO_2_ cooling is thinner than that of water cooling. This is due to the decrease in cooling media temperature, peak temperature, and residence time at a high temperature decrease, inhibiting austenite coarsening. In this case, the fine austenite phase transforms into fine lath martensite.

### 3.2. Mechanical Properties of the Joints under Various Cooling Media

**Figure 12:**
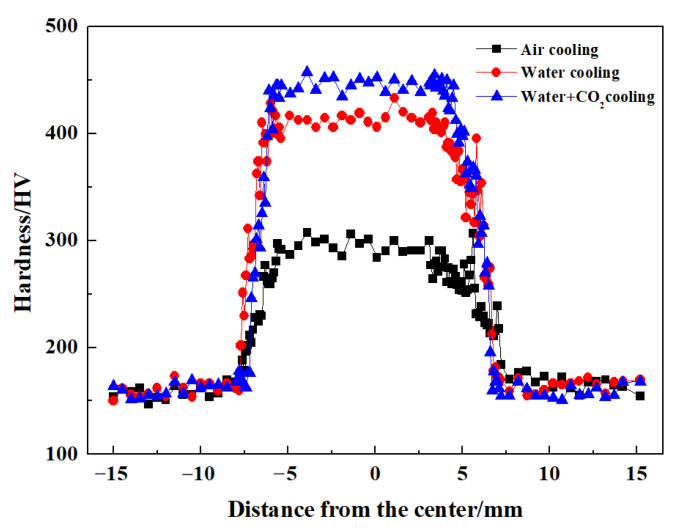
Vickers hardness distribution profile of joints under various cooling media.

Figure 12 presents the Vickers hardness in FSW cryogenic steel joints under various cooling media. The lowest hardness of ~150 HV was found in the BM because of the appearance of ferrite and pearlite. Under air-cooling conditions, the hardness in the joint increases gradually near NZ. Finally, the NZ shows the highest hardness value of ~295 HV. The hardness changes in joints under water-cooling and water + CO_2_-cooling conditions are similar to those under air-cooling conditions. However, the hardness value in the NZ increases significantly when decreasing the cooling media temperature. Some studies [14,15] suggested that the hardness of the joint was mainly affected by grain size and phase-transformed products. Under air-cooling conditions, near the NZ, the microstructure in the HAZ gradually changes from pearlite + ferrite to bainite + martensite, increasing hardness. The NZ consisting of martensite + bainite presented high hardness. Furthermore, martensite content in the joint increases when decreasing the cooling media temperature, improving the hardness. Accordingly, the NZ under water + CO_2_-cooling condition containing fine martensite microstructure had the highest hardness value of ~445 HV. By comparison, the lowest-hardness regions were found in the SCHAZ of FSW martensitic steel owing to tempering softening of martensite at a peak temperature lower than A_c1_ for a long time [16]. In this study, there is no obvious softening of the HAZ in FSW cryogenic steel because the presence of bainite and martensite inhibited the decrease in hardness.

**Figure 13:**
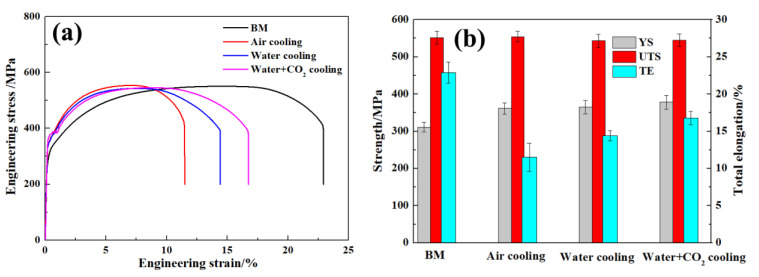
Tensile properties of the joints under various cooling media and BM. (**a**) Engineering stress–strain curves, (**b**) specific tensile properties.

Figure 13a exhibits engineering stress–strain curves of the joints under different cooling media and BM. The ultimate tensile strength (UTS), total elongation (TL), and yield strength (YS) in the BM were 550 MPa, 22.8%, and 310 MPa, respectively, as shown in Figure 13a,b. The tensile fracturing of the joint under various cooling media occurred in the BM with low hardness. Under air-cooling conditions, YS, UTS, and TL of the joint were 361 MPa, 554 MPa, and 11.5%, respectively. The UTS of the joints under various cooling media was similar to that of the BM, which coincided with the fracture location of the joints occurring in the BM. However, the YS and TL of the joints increased as the temperature of the cooling media decreased. Ultimately, the joints with excellent combinations of UTS of 545 MPa and TL of 16.8% were obtained under water + CO_2_-cooling conditions.

As the temperature of the cooling media decreased, the grain of the joints was significantly refined and the amount of martensite increased, leading to an increase in the YS of the joints. In addition, compared to the coarse bainite + martensite microstructure in the joint under air-cooling conditions, the fine martensite microstructure in the joints under water + CO_2_-cooling conditions enhanced the plastic deformation capacity of the joints. In contrast, Prajapati et al. [2] suggested that the UTS and TL of a gas metal arc welded (GMAW) cryogenic steel joint attained values 84% and 41% of those of the BM, respectively. Obviously, the FSW cryogenic steel joint under external rapid cooling conditions exhibited an excellent tensile property.

Table 1 exhibits the impact energy of the HAZ and NZ under different cooling media and the BM. The impact energies in the HAZ under air cooling, water cooling, and water + CO_2_ cooling were 85, 90, and 95 J/cm^2^, respectively, while those of the NZs were 88, 98, and 110 J/cm^2^, respectively. Apparently, the toughness in the HAZ and NZ increases when decreasing cooling media temperature. The good toughness in the HAZ and NZ under the water + CO_2_-cooling conditions reaches 83% and 96% of that of the BM, respectively. Usually, the toughness of microalloy steels is mainly determined by microstructural characteristics, grain boundary, and grain size. Moreover, previous studies show bainite or martensite packets having HAB impede crack propagation [17]. When the crack meets the HAB, the crack propagation direction is deflected, thereby improving the toughness. In this study, as the cooling media temperature decreases, the grain in the HAZ and NZ is refined and a high fraction of HAB is obtained, thus improving the toughness. Therefore, the joint under the water + CO_2_-cooling condition shows high toughness. By comparison, Teske et al. [3] performed GMAW on cryogenic steel and pointed out that the joint exhibited a low impact energy of 19 J. Based on the above analysis, external rapid cooling media can effectively enhance the mechanical properties of FSW cryogenic steel joints.

**Figure 14:**
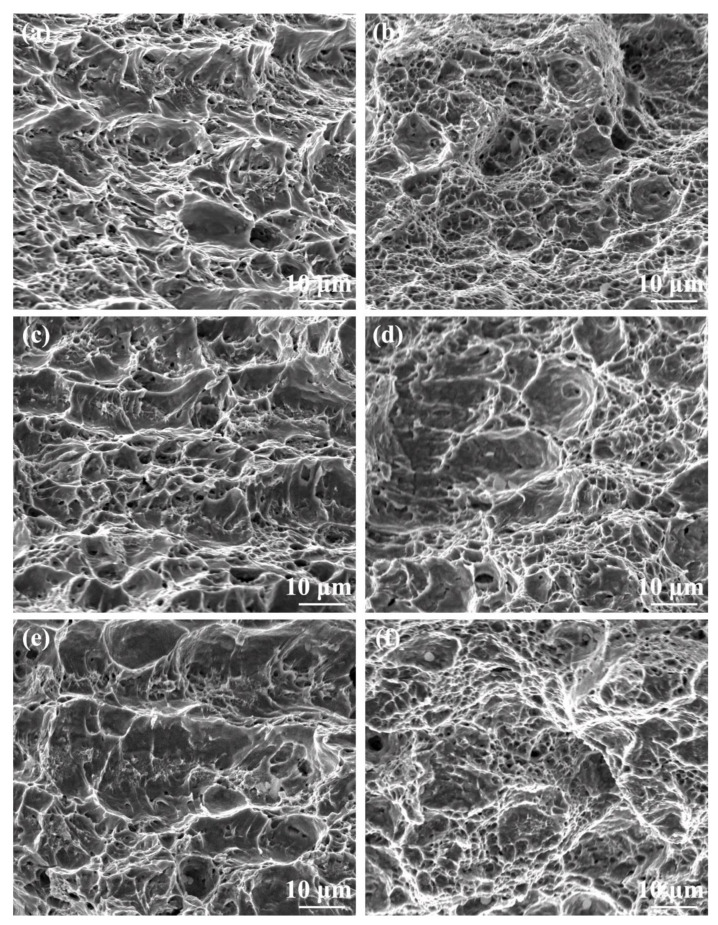
SEM images of impact fractural surface in the HAZ and NZ under various cooling media. (**a**,**b**) Air cooling, (**c**,**d**) water cooling, (**e**,**f**) water + CO_2_ cooling, (**a**,**c**,**e**) HAZ, (**b**,**d**,**f**) NZ.

Figure 14 shows the SEM images of the impact fractural surfaces in the HAZ and NZ under various cooling media. A large number of deep dimples and apparent tearing edges were detected on the fracture surfaces of the NZ under water + CO_2_-cooling conditions (Figure 14f), indicating excellent ductile fracture characteristics. With the increase in the cooling media temperature, the dimples were shallow. Compared with the NZ, the number of tiny dimples in the HAZ noticeably decreased. With the increase in the cooling media temperature, the number of dimples decreases, resulting in lower toughness.

## 4. Conclusions

In this study, FSW was used to connect 2.5 mm thick cryogenic steels under various cooling media of air, water, and water + CO_2_. The primary results are as follows:

(1) The joint under various cooling media presented a basin and had no obvious defects. Under air-cooling conditions, the SCHAZ contained ferrite + pearlite, the ICHAZ consisted of a mixed microstructure of ferrite + pearlite + bainite + martensite, and the FGHAZ and NZ contained bainite + martensite. Martensite content in the joints increases with the decrease in the cooling media temperature because of the larger undercooling degree.
(2) Under air-cooling conditions, the hardness in the joint increases gradually near the NZ, and the NZ shows the highest hardness value. As the cooling media temperature decreased, the hardness in the NZ increased significantly when decreasing cooling media temperature.
(3) The tensile fracture of the joint occurred in the BM, and with decreasing cooling media temperature, the YS and TL of the joint increased obviously. A remarkable combination of strength (545 MPa) and elongation (16.8%) was obtained in the joint under water + CO_2_-cooling conditions.
(4) As the cooling media temperature decreases, the toughness in the NZ and HAZ is improved. The good toughness in the HAZ and NZ under water + CO_2_-cooling conditions reaches 83% and 96% of that of the BM, respectively.

## Figures and Tables

**Table 1:** Impact energy of joint under various cooling media at −46 °C (J/cm^2^).

V-Shaped Notch Locations	HAZ	NZ	BM
Air cooling	85 ± 7	88 ± 7	115 ± 5
Water cooling	90 ± 4	98 ± 9	115 ± 5
Water + CO_2_ cooling	95 ± 4	110 ± 7	115 ± 5

## Data Availability

Data are contained within the article
